# Long-term cerebral thromboembolic complications of transapical endocardial resynchronization therapy

**DOI:** 10.1007/s10840-016-0206-6

**Published:** 2016-11-12

**Authors:** Zsuzsanna Kis, Andrea Arany, Gabriella Gyori, Attila Mihalcz, Attila Kardos, Csaba Foldesi, Imre Kassai, Tamas Szili-Torok

**Affiliations:** 1 Gottsegen György National Institute of Cardiology, Haller utca 29, 1094 Budapest, Hungary; 2 United St Istvan and St Laszlo Hospital, Nagyvarad ter 1., 1097 Budapest, Hungary; 3 Thoraxcenter, Department of Clinical Electrophysiology, Erasmus MC, ‘s Gravendijkwal 230, Kamer BD416, Postbus 2040, 3000 CA Rotterdam, The Netherlands

**Keywords:** Transapical left ventricle pacing, Resynchronization therapy, End-stage heart failure, Thromboembolic complication

## Abstract

**Purpose:**

Cardiac resynchronization therapy (CRT) is an established therapeutic option in selected heart failure patients (pts). However, the transvenous left ventricular (LV) lead implantation remains ineffectual in a considerable number of pts. Transapical LV (TALV) lead implantation is an alternative minimally invasive, surgical, endocardial implantation technique. The aim of the present prospective study is to determine the long-term outcome, including the cerebral thromboembolic complications, of pts who underwent TALV lead placement.

**Methods:**

Twenty-six CRT candidates (19 men (78 %); mean age 61 ± 10 years) with a previously failed transvenous approach underwent TALV lead placement as a last resort therapy. The following data was collected: mortality rate, reoperation rate, and cerebrovascular event rate. Patients underwent a cerebral CT scan to determine any possible cerebrovascular event related to the presence of the TALV lead.

**Results:**

Eleven out of 26 (47 %) patients survived after a median follow-up of 40 ± 24.5 months. Major acute ischemic stroke occurred in two cases, while in one case transient ischemic stroke was observed. Cerebral CT scan examination performed in asymptomatic patients revealed chronic ischemic lesions with minimal extension in two patients. Reoperation occurred in one case due to TALV lead fracture.

**Conclusions:**

This is the first study reporting the long-term outcome, mortality, and thromboembolic event rate exclusively after TALV lead implantation. Patients who underwent TALV lead implantation have a comparable long-term mortality rate to conventional CRT, although a major ischemic cerebrovascular event after TALV lead implantation is worrisome and has an impact on the outcome.

## Introduction

Cardiac resynchronization therapy (CRT) is an established therapeutic option in a subgroup of heart failure patients, which improves heart function and functional clinical status and decreases mortality [1–3]. Despite significant technological improvements, the transvenous left ventricular (LV) pacing lead implantation into one of the branches of the coronary sinus (CS) can remain ineffectual in certain cases [4, 5]. Epicardial pacing lead implantation is the most frequently used alternative, although this requires open heart surgery [6]. Furthermore, reaching and pacing the most delayed LV segment can be challenging due to LV dilatation. In addition, the location of the epicardial coronaries may be obscured due to pericardial adhesions. Consequently, the prevention of any damage to important vessels while inserting the epicardial LV lead can be difficult. Nevertheless, epicardial pacing seems to be less effective compared to endocardial LV pacing [7]. Endocardial stimulation is associated with a greater aortic and mitral time velocity integral, and increased left ventricular fractional shortening in comparison with epicardial stimulation [7]. Endocardial left ventricular pacing can be achieved by different techniques such as transseptal and transapical left ventricular pacing approaches. Transseptal cardiac resynchronization therapy carries a high risk of device-related infective endocarditis. This condition can only be treated by hazardous surgical lead extraction and repair or replacement of the mitral valve when affected [8, 9]. The transapical left ventricular (TALV) lead implantation technique can eliminate most of the aforementioned problems. The main advantages of the transapical technique are the following: a minimally invasive, surgical technique ensuring endocardial LV stimulation, avoiding damage caused by contact with the mitral valve, which provides an alternative last resort therapy for severely affected patients [8, 9].

Despite promising mid-term transapical CRT outcome data, recent evidence raised concerns about the long-term thromboembolic complications of endocardial LV pacing techniques [10]. The aim of the present single-center prospective study was to assess the long-term outcome and the incidence of thromboembolic complications in patients who underwent transapical endocardial LV lead placement.

## Methods

### Patient population

This study was approved by the Regional Medical Ethical Committee conform the Medical Research Council-Scientific and Ethical Committee guidelines of the 1975 Declaration of Helsinki. Informed consent was obtained from all patients before the procedure. Between October 2007 and September 2013, 26 consecutive patients (mean age 61 ± 10; seven women) with ischemic (12 pts) and dilated (14 pts) cardiomyopathy after failed transvenous LV lead implantation underwent TALV lead placement as a last resort therapy. All patients were eligible for CRT according to the ACC/AHA/ESC guidelines [11, 12]. The main demographic data and the medical therapy of patients are summarized in Table 1. Since lifelong anticoagulation therapy is mandatory after left ventricle lead implantation, patients with any contraindication to anticoagulation therapy were excluded from the study. Further exclusion criteria were the following: presence of intracavital thrombus, preoperative pericardial effusion, and/ or large coronary artery branches around the apex [13–15].

**Table 1:** Baseline clinical and demographic characteristics

Parameter	Mean ± SD or %
Age at enrolment (years)	61 ± 10
Sex	
Male	19 (73 %)
Female	7 (27 %)
Cardiomyopathy	
Dilated cardiomyopathy(DCM)	14 (54 %)
Ischemic cardiomyopathy (ICM)	12 (46 %)
New York Heart Association functional class (NYHA)	
II.	2 (8 %)
III.	17(65 %)
IV.	7 (27 %)
Left ventricle ejection fraction at enrolment (LVEF %)	26.7 ± 6.63
Left ventricle end-systolic diameter at enrolment (LVESD, mm)	75.08 ± 17.15
Left ventricle end-diastolic diameter et enrolment (LVEDD, mm)	62.56 ± 11.62
Intrinsic QRS duration (ms) at enrolment	167.85 ± 24.05
Rhythm at time of implantation	
Sinus rhythm	21/26
Atrial fibrillation	3/26
Pm rhythm	2/26
Drug therapy	
ACE inhibitors, ARBs (yes/no [% of yes])	21/26 (80 %)
Beta-blockers (yes/no [% of yes])	21/26 (80 %)
Digoxin (yes/no [% of yes])	6/26 (23 %)
Amiodarone (yes/no [% of yes])	9/26 (34 %)
Loop diuretics (yes/no [% of yes])	20/26 (77 %)
Spironolactone (yes/no [% of yes])	15/26 (57 %)
Procedural data	
Operation time (min)	65 ± 14
Fluoroscopy time (min)	3,6 ± 0,8
Postoperative stay in hospital (day)	9,5 ± 5

*SD* standard deviation, *ACE* angiotensin convertase enzyme, *ARB* angiotensin receptor Blocker

### Surgical procedures

**Fig. 1:**
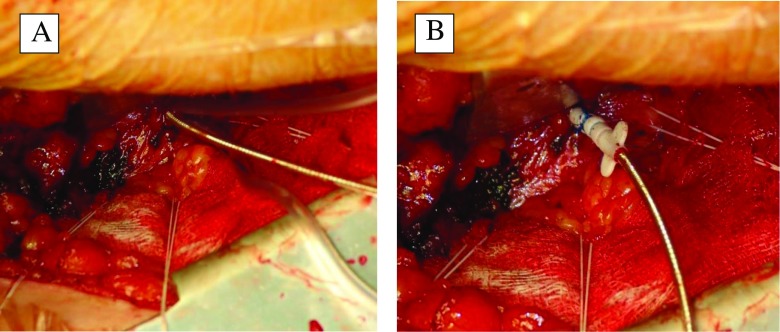
Intraoperative photo of the transapical left ventricle lead insertion and fixation. **a** Puncture and dilatation of the left ventricle apex using Seldinger-technique. **b** Fixation of the transapical left ventricular lead using purse-string suture around the puncture site

**Fig. 2:**
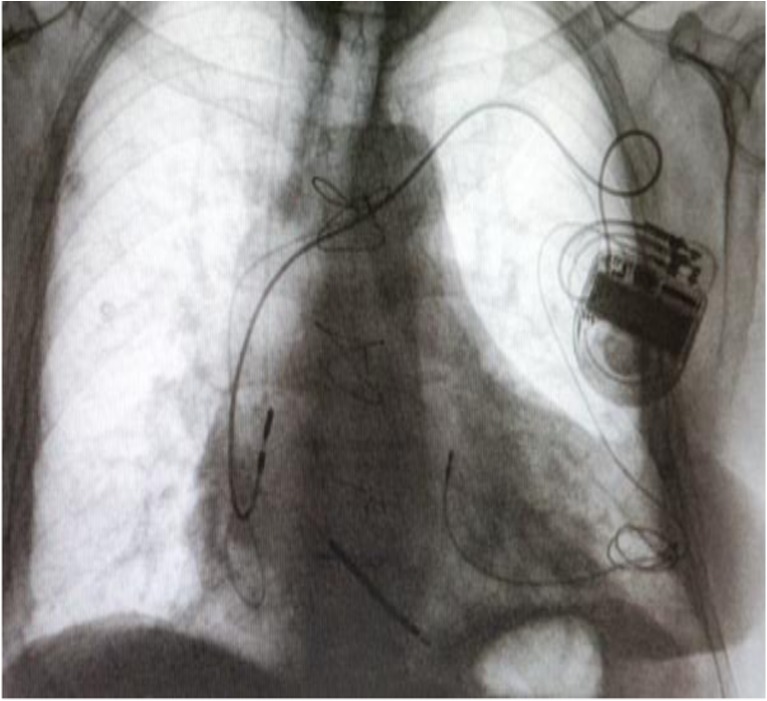
Positioning and fixation of transapical left ventricular lead under fluoroscopy guidance

The method of TALV lead implantation has been previously reported [8, 9]. The procedures were performed under general anesthesia. The patients were positioned for a limited left thoracotomy via an infraclavicular incision. Initially, transthoracic echocardiography was used to locate the LV apex. By means of the mini-thoracotomy, a pericardiotomy was executed above the LV apex to ensure free navigation of the LV lead. Any type of active fixation pacing lead was allowed for insertion through the apex into the LV cavity. We preferred the use of the thinnest bipolar electrodes to decrease the traumatic effect during insertion. A standard Seldinger-technique with a peel-away sheath was used for insertion. Firstly, the apex was punctured with a needle and a guidewire inserted through the needle. After removal of the needle the apex puncture was dilated with a peel-away sheath and dilator placed over the guide wire. The guidewire and dilator were removed and the pacing electrode inserted into the LV cavity. Once the lead was in position, the peel-away sheath was removed. A monofilament purse-string suture was applied around the insertion site to control hemorrhage from the LV cavity (Fig. 1). Navigation and endocardial fixation of the LV lead was performed under fluoroscopic guidance (Fig. 2). After appropriate endocardial LV lead-fixation, pacing and sensing measurements were performed. The acceptable pacing threshold was less than 1 V and R-wave amplitude for sensing in this electrode was more than 5 mV. Purse-string sutures were used employed at the apex to minimize the electrode movement through the apex and they also were attached to the body of the electrode to achieve a stable position [9]. Afterwards, the proximal portion of the electrode was subcutaneously tunneled to the infraclavicular area and connected to the CRT-device [14]. The right atrial and ventricular leads were implanted through the cephalic or subclavian vein using traditional percutaneous technique [8, 14].

Finally, a pleural drain was inserted followed by standard wound closure. The perioperative anticoagulation protocol was identical to patients who underwent mitral valve replacement with mechanical valve prosthesis [16]. Intravenous heparin was started 3 h postoperatively in the absence of bleeding from pleural drain. Oral anticoagulation therapy was designed to reach the targeted INR level (2.5–3.5) bridging with heparin. Procedural data are summarized in Table 2.

**Table 2:** Type of CRT devices and TALV leads

Type of CRT devices	Number (*n* = 26)
Biotronik Lumax	6
Biotronik Stratos	8
Biotronik Entvios	1
Medtronic Syncra	1
Medtronic Insyc/Concerto	7
St. Jude Atlas/Promote	2
Boston Scientific Cognis	1
Type of TALV leads	Number (*n* = 26)
Vitatron ICQ09B	4
Giant Flextend2	1
St. Jude 1888T	8
Medtronic 5076	7
Medtronic 6944	1
Medtronic 4076	5

*CRT* cardiac resynchronization therapy, *TALV* transapical left ventricular

### Device implantation and pacing mode

Before surgical LV lead implantation, to identify the optimal LV pacing site, the most delayed LV segment was determined by tissue Doppler imaging and/or by electrical activation with electroanatomical mapping [14]. Twenty-six patients received CRT devices, and the pacing algorithm was biventricular DDD mode. Twelve patients underwent CRT-PM implantation while in fourteen patients CRT-D device implantation was performed. The majority of the patients were in sinus rhythm at the time of implantation. The interventricular (VV) time was empirically defined as minus 20 ms (LV first) [14]. The type of CRT devices and the type of TALV leads are described in Table 1.

### Follow-up and cerebral CT scan

All patients were scheduled for regular visits at 1, 3, 6 months and every 6 months after that. Additional visits or hospitalizations were registered. The INR level was checked and corrected to be in the range between 2.5 and 3.5 generally monthly but if required daily. During the median follow-up period of 40 ± 24.5 months, we collected data on mortality rate, reoperation rate, and cerebrovascular event rate. Emergency CT scan was performed in patients with symptomatic and/or suspected ischemic thromboembolic event.

Asymptomatic patients underwent an elective, non-contrast enhanced cerebral CT scan examination at median follow-up of 40 ± 24.5 months in order to determine any silent thromboembolic event possibly related to the presence of the LV endocardial lead.

Scans were performed using a Siemens Somatom Sensation 40 CT scanner. The scanning parameters were 140 kV and 230 mA. Estimated effective radiation dose was 2.2 mSv (average DLP 1092 mGy cm). The CT scan enabled the acquisition of 40 slices per rotation with a 2-mm slice width.

### Statistical analysis

Descriptive statistics were performed. Continuous variables were presented as mean ± standard deviation (SD) and compared with Student’s *t* test. Categorical data were expressed in percentage.

## Results

### Mortality rate

During the median follow-up period of 40 ± 24.5 months, 3 out of 26 patients with transapical CRT were crossed over to epicardial LV lead implantation; consequently, 23 pts could be followed-up as pts with TALV lead implantation. The mortality rate was determined utilizing the National Registry Office database. Eleven out of 23 (47 %) patients with transapical CRT survived after a median follow-up of 40 ± 24.5 months. One patient was lost to follow-up. Ten patients died due to exacerbated heart failure while one patient suffered sudden cardiac death.

### Morbidity rate

Two out of the three patients crossed over to an epicardial CRT system underwent right-sided infective endocarditis. In the first case, the infection occurred 3 months after the TALV lead implantation procedure. The second case materialized 3 years after the necessity of TALV lead repositioning and reoperation, CRT generator decubitus was diagnosed. In these cases, a new epicardial CRT-system was implanted via medial sternotomy accompanied by the administration of antibiotic-therapy. A third patient was admitted to our hospital 1 month after the transapical CRT implantation with symptoms of pericardial tamponade, caused by the dislocation of the TALV lead. During an emergency reoperation, the transapical LV lead was removed and a new epicardial LV lead placed. Furthermore, two cases of CRT-pocket infection were observed and two cases CRT-pocket hematoma.

### Procedural data

*Reimplantation* was necessary in one patient, after interruption of anticoagulation therapy, due to TALV lead fracture causing the deterioration of heart failure, 5 years after the primary procedure.

*Repositioning* of the TALV lead was necessary in three cases. In two patients, lead dislocation was detected before discharge from hospital. In one of the cases, it occurred during closure of the pericardium, while in the other case, it was observed on the second postoperative day [8]. Repositioning of these electrodes was performed without re-opening the pleural cavity [8]. In one case, TALV lead repositioning had to be performed due to lack of capture at maximal output (7.5 V /1.5 ms) despite repeated programming attempts.

In another patient, 1 week after the transapical CRT implantation, dislocation of the right atrial electrode was observed. In one other case, deterioration of heart failure was detected, caused by right ventricular lead dislocation. Both cases were resolved by repositioning of the dislocated electrodes. In yet another patient, a local pocket infection was detected, 2 years after the TALV lead implantation, requiring CRT-P generator repositioning. Procedural complications are summarized in Table 3. Dislocation of the TALV leads can possibly be explained by different mechanisms. One of the suspected mechanisms of TALV lead dislocation is incomplete screw-in and subsequent tip release from the endocardium [8]. The other possible mechanism of TALV lead dislocation may derive from the favorable changes in LV function after CRT. Since better LV function and the more effective contraction are achieved with CRT, this may increase the chance of the LV lead pulling out [8]. To avoid this complication, as we previously reported, the lead should be securely fixed at the apex and its position checked by chest X-ray 3–5 weeks after the implantation [13]. Despite all the procedures were performed by a highly experienced operator, the lack of a dedicated device such as the learning curve of this novel implantation technique might explain the slightly high complication rate.

**Table 3:** Procedural complication

Reoperation needed *n* = 4
-1 reoperation due to TALV lead fracture
-2 reoperations due to right-sided infective endocarditis
-1 reoperation due to TALV lead dislocation
Reposition needed *n* = 6
-2 repositioning due to TALV lead dislocation
-1 repositioning due to TALV lead capture problem
-1 repositioning due to right atrial lead dislocation
-1 repositioning due to right ventricle lead dislocation
-1 device generator repositioning due to pocket infection
Hematoma *n* = 2
Pocket infection *n* = 2

*TALV* transapical left ventricle

### Thromboembolic complications and cerebral CT scan

The coexisting atrial fibrillation may increase the risk of thromboembolic events. Atrial fibrillation was observed in three patients at the time of device implantation. However, during the follow-up period, atrial fibrillation was detected in ten out of 26 patients. We chose CT scan instead of magnetic resonance imaging (MRI) modality to detect evidence of an ischemic event as neither the CRT devices nor the attached leads were MRI compatible. During the follow-up period, one case of right-sided hemiplegia was observed 2 months after the TALV lead implantation. An urgent non-contrast enhanced cerebral CT scan identified an acute ischemic occlusion in the middle cerebral artery. Systemic thrombolytic therapy could not be applied as the patient was receiving effective anticoagulation therapy. This was the second ischemic stroke, with signs of right-sided hemiplegia, that the patient had suffered.

**Fig. 3:**
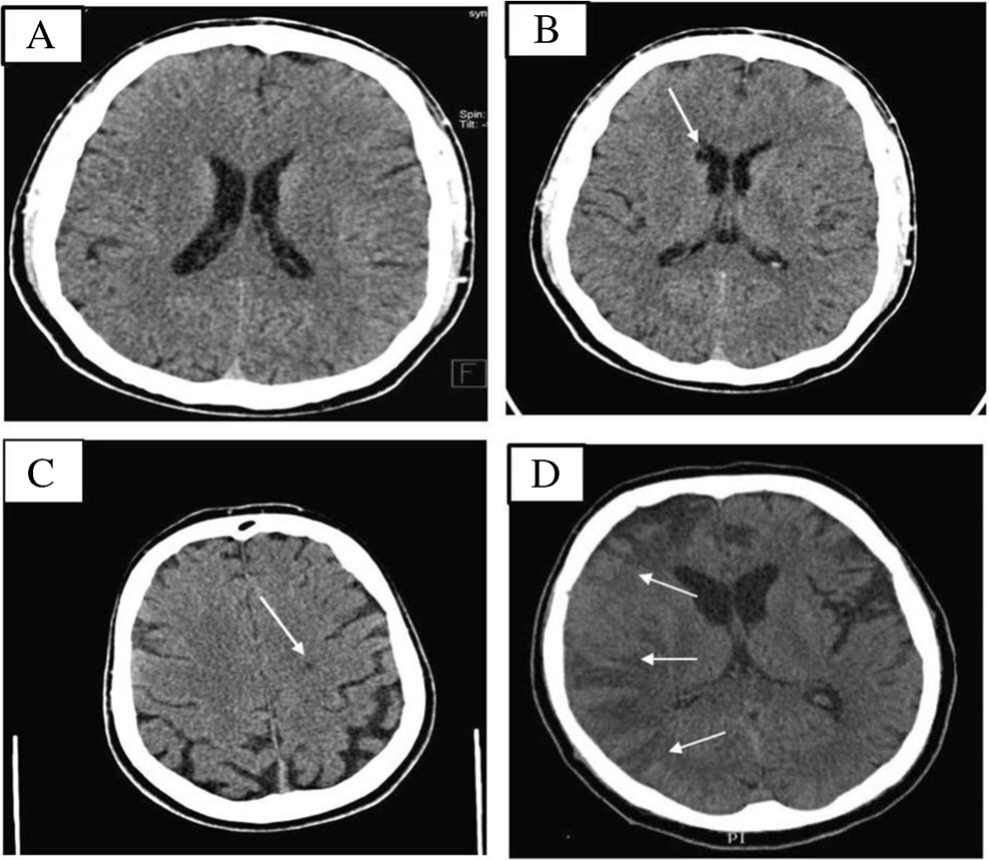
Non-contrast enhanced cerebral CT scan of patients after TALV lead implantation: **a** no abnormality; **b** 6 mm lacuna in the right-sided nucleus caudatus, **c** 4 mm hypodensity in left-sided centrum semiovale, **d** middle cerebral artery occlusion with right-sided fronto-temporo-parietale extension

There was an earlier occurrence 6 years before TALV implantation. Both of these ischemic events healed without any clinical symptoms. This patient died 3 years after the TALV implantation due to heart failure deterioration. In the patient who underwent reoperation due to TALV lead fracture, requiring interruption of the anticoagulation therapy, left-sided hemiparesis occurred 3 days after the procedure. The urgent CT scan examination revealed acute major right-sided middle cerebral artery occlusion with fronto-temporo-parietale extension (Fig. 3d). Thrombolytic therapy was contraindicated because of the history of anticoagulation therapy and the CRT-device reoperation within 1 week of this occurrence. The patient received conservative therapy and neurological rehabilitation with good success. In one case, facio-brachial predominant hemiparesis occurred 4 months after TALV lead placement. The CT scan revealed bilateral chronic ischemic stroke; however, an acute lesion could not be detected. Thrombolytic therapy was not instituted because of the absence of an acute ischemic lesion and the presence of continuing effective anticoagulation therapy. The patient’s symptoms resolved after the administration of high dose parenteral vasoactive medication. Nine months after TALV lead implantation, successful LVAD implantation was performed.

In asymptomatic patients, the CT scan examination, performed at the median follow-up of 40 ± 24.5 months, revealed minimal extension chronic ischemic lesions in two cases (6 mm lacuna in the right-sided nucleus caudatus, 4 mm hypodensity in the left-sided centrum semiovale) (Fig. 3b, c).

## Discussion

The major finding of this study is that, although transapical CRT can be used as an alternative method for CRT in selected heart failure patients, it represents a worrisome thromboembolic complication rate compared to traditional transvenous CRT. As we previously reported, patients after TALV lead implantation with 18-months follow-up period presented promising outcomes with potential advantages such as shorter procedure time and decreased postoperative burden compared to epicardial left ventricle lead implantation techniques. [14] However, only a few reports dealt with the thromboembolic complications of LV endocardial pacing. Jais et al. and Pasquie et al. with a clinical follow-up of 15 ± 12 and 85 ± 5 months, both reported transient ischemic attack (TIA), 1 out of 11 and 1 out of 6 patients [17, 18]. Rademakers et al. investigated the thromboembolic complication of endocardial LV lead pacing (45 transseptal, 6 transapical) with mid-term follow-up [10]. The incidence of thromboembolic events per 100 patient-years was 6.1. Five patients had an ischemic stroke (one had both stroke and TIA) and two patients suffered from TIA [10]. In these cases, the thromboembolic events happened after interruption of anticoagulation therapy [10]. In our study, two major stroke and one transient ischemic attack occurred during median follow-up of 40 ± 24.5 months. One out of two thromboembolic events happened early after the interruption of anticoagulation therapy due to the necessity of TALV lead reoperation. Consequently, the major cerebrovascular events were probably associated with insufficient anticoagulation levels as stated in the reports of Jais et al. and Pasquie et al. [17, 18]. The short-term cerebral thromboembolic complications might be lowered if anticoagulation therapy would not be interrupted with INR kept at >2. Subtherapeutic INR levels frequently appear in everyday practice [10]. According to previous studies, only two thirds of patients are within the target INR level. The duration of decreased anticoagulation control is associated with increased risk of stroke [19]. Despite the fact that the efficacy of the novel oral anticoagulants is more predictable, no experience with its use is available in the endocardial LV pacing patient population. Chronic heart failure and left ventricular dilatation represents a higher risk of thromboembolism [20]. The severity of decreased ejection fraction appears to be an independent risk factor for thromboembolic events in women [21]. In the SAVE trial, the risk of stroke was nearly twice as high among patients with LVEF under 28 % than in the control group (LVEF > 29 %) after myocardial infarction [22]. Lead components may also influence the risk of stroke. The thrombogenicity of polyurethane leads may be lower than those of silicone [23]. The report of Rademakers et al. investigating cerebral thromboembolic complications after endocardial lead placement (45 atrial transseptal, 6 transapical) showed that all events happened with smaller diameter select secure leads which had the same polyurethane outer insulation [10]. This result makes unlikely that the outer insulation of endocardial LV lead is a critical factor in stroke occurrence [10]. The presence of an intraventricular anodal electrode may represent an unknown factor as the source of intracavital thrombus formation. The movement of the TALV electrode may generate increased turbulent blood flow in the left ventricle generating thrombus formation. Nowadays, novel therapeutic options should be involved widely in the therapeutic regime of end-stage heart failure patients. The application of left ventricular or biventricular assist devices could be used as destination therapy in end-stage heart failure patients; however, one of their major complications is the occurrence of thromboembolic events.

Baroreflex activation therapy with centrally mediated reduction of sympathetic outflow and increased parasympathetic activity results in improvement of functional status, quality of life, and exercise capacity. The technique can also be advised for heart failure patients; however, its long-term outcome is still unknown [24].

### Limitations of the study

The lack of a control group does not make it possible to determine the contribution of left ventricular endocardial lead implantation to the occurrence of thromboembolic events. A larger patient population is needed to be able to compare the thromboembolic complications of transapical LV lead implantation to traditional transvenous or epicardial LV lead positioning approaches. A further limitation of the study is the relatively small number of implantations, which can be attributed to the very strict inclusion criteria. In the case of device or TALV lead endocarditis, in the absence of special extraction techniques, the only solution to remove the CRT-system is reoperation via sternotomy, which is a high-risk maneuver in this severely diseased patient population. Additionally, the absence of utilizing brain MRI scan to detect evidence of a cerebral ischemic event is a definite limitation of this study. As neither MRI-compatible leads nor CRT devices were available during the enrollment period, brain MRI scan was not the method of choice to be executed during the follow-up period. Consequently, it is possible that the presence of silent cerebral thromboembolic lesions likely related to TALV lead implantation is underestimated.

## Conclusions

In conclusion, this study reports on long-term outcome and mortality rate after implantation of a transapical left ventricular endocardial lead. Patients who underwent TALV lead implantation have a reasonable long-term mortality rate, although occurrence of major ischemic cerebrovascular event after transapical LV lead implantation is worrisome and has an impact on the outcome. Based on the aforementioned findings, which are in accordance with other reported results, the value of endocardial left ventricular pacing is questionable and consequently cannot be promoted as an alternative technique for CRT. It can only be considered in selected cases. Theoretically, an improved anticoagulation strategy, either using novel anticoagulants or ensuring a higher target-INR level with standard anticoagulants, might lower the occurrence of thromboembolic events. This should obviously be tested in prospective trials.

## Abbreviation

CRT: Cardiac resynchronization therapy
CS: Coronary sinus
CT scan: Computer tomography scan
INR: International normalized ratio
kV: Kilovolt
LV: Left ventricle
LVEDD: Left ventricular end-diastolic diameter
LVEF: Left ventricular ejection fraction
mA: Mili-amper
mGy: Mili-Gray
MRI: Magnetic resonance imaging
mSv: Mili-Sievert
NYHA classification: New York Heart Association classification
TALV lead: Transapical left ventricular lead
TEE: Transesophageal echocardiography

## References

[1] Cleland JGF, Daubert J-C, Erdmann E, for the Cardiac Resynchronization-Heart Failure (CARE-HF) Study Investigators (2005). The effect of cardiac resynchronization on morbidity and mortality in heart failure. N Engl J Med.

[2] Cazeau S, Leclercq C, Lavergne T, for the Multisite Stimulation in Cardiomyopathies (MUSTIC) Study Investigators (2001). Effects of multisite biventricular pacing in patients with heart failure and intraventricular conduction delay. N Engl J Med.

[3] Abraham WT, Fisher WG, Smith AL, for the MIRACLE Study Group (2002). Cardiac resynchronization in chronic heart failure. N Engl J Med.

[4] León AR, Abraham WT, Curtis AB, MIRACLE Study Program (2005). Safety of transvenous cardiac resynchronization system implantation in patients with chronic heart failure: combined results of over 2,000 patients from a multicentre study program. J Am Coll Cardiol.

[5] Gassis SA, Delurgio DB, Leon AR (2006). Progress in cardiovascular disease: technical considerations in cardiac resynchronization therapy. Prog Cardiovasc Dis.

[6] Puglisi A, Lunati M, Marullo AG (2004). Limited thoracotomy as a second choice alternative to transvenous implant for cardiac resynchronisation therapy delivery. Eur Heart J.

[7] van Gelder BM, Scheffer MG, Meijer A (2007). Transseptal endocardial left ventricular pacing: an alternative technique for coronary sinus lead placement in cardiac resynchronization therapy. Heart Rhythm.

[8] Kassai I, Mihalcz A, Foldesi C (2009). A novel approach for endocardial resynchronization therapy: initial experience with transapical implantation of the left ventricular lead. Heart Surg Forum.

[9] Kassai I, Foldesi C, Szekely A (2009). Alternative method for cardiac resynchronization: transapical lead implantation. Ann Thorac Surg.

[10] Rademakers LM, van Gelder BM, Scheffer MG (2014). Mid-term follow up of thromboembolic complications in left ventricular endocardial cardiac resynchronization therapy. Heart Rhythm.

[11] 2012 ACCF/AHA/HRS Focused Update Incorporated Into the ACCF/AHA/HRS 2008 Guidelines for device-based therapy of cardiac rhythm abnormalities: a report of the American College of Cardiology Foundation/American Heart Association Task Force on Practice Guidelines and the Heart Rhythm Society. J Am Coll Cardiol. 2013;61(3):e6–75. doi:10.1016/j.jacc.2012.11.007. 10.1016/j.jacc.2012.11.007 23265327

[12] 2013 ESC Guidelines on cardiac pacing and cardiac resynchronization therapy. The Task Force on cardiac pacing and resynchronization therapy of the European Society of Cardiology (ESC). Developed in collaboration with the European Heart Rhythm Association (EHRA). Eur Heart J. 2013;34:2281–329. doi:10.1093/eurheartj/eht150. 10.1093/eurheartj/eht150 23801822

[13] Kassai I, Friedrich O, Ratnatunga C (2011). Feasibility of percutaneous implantation of transapical endocardial left ventricular pacing electrode for cardiac resynchronization therapy. Europace.

[14] Mihalcz A, Kassai I, Kardos A (2012). Comparison of the efficacy of two surgical alternatives for cardiac resynchronization therapy: trans-apical versus epicardial left ventricular pacing. Pacing Clin Electrophysiol.

[15] Kassai I, Foldesi C, Szekely A (2008). New method for cardiac resynchronization therapy: transapical endocardial lead implantation for left ventricular free wall pacing. Europace.

[16] Vahanian A, Alfieri O, Andreotti F (2012). Guidelines on the management of valvular heart disease (version 2012). The Joint Task Force on the Management of Valvular Heart Disease of the European Society of Cardiology (ESC) and the European Association for Cardio-Thoracic Surgery (EACTS). Eur Heart J.

[17] Jaïs P, Takahashi A, Garrigue S (2000). Mid-term follow-up of endocardial biventricular pacing. Pacing Clin Electrophysiol.

[18] Pasquié JL, Massin F, Macia JC (2007). Long-term follow-up of biventricular pacing using a totally endocardial approach in patients with end-stage cardiac failure. Pacing Clin Electrophysiol.

[19] Gallagher AM, Setakis E, Plumb JM (2011). Risks of stroke and mortality associated with suboptimal anticoagulation in atrial fibrillation patients. Thromb Haemost.

[20] Sirajuddin RA, Miller AB, Geraci SA (2002). Anticoagulation in patients with dilated cardiomyopathy and sinus rhythm: a critical literature review. J Card Fail.

[21] Dries DL, Rosenberg YD, Waclawiw MA (1997). Ejection fraction and risk of thromboembolic events in patients with systolic dysfunction and sinus rhythm: evidence for gender differences in the studies of left ventricular dysfunction trials. J Am Coll Cardiol.

[22] Loh E, Sutton MSJ, Wun C-CC (1997). Ventricular dysfunction and the risk of stroke after myocardial infarction. N Engl J Med.

[23] Palatianos GM, Dewanjee MK, Panoutsopoulos G (1994). Comparative thrombogenicity of pacemaker leads. Pacing Clin Electrophysiol.

[24] Abraham WT, Zile MR, Weaver FA (2015). Baroreflex activation therapy for the treatment of heart failure with a reduced ejection fraction. JACC Heart Fail.

